# Role of PARP in TNBC: Mechanism of Inhibition, Clinical Applications, and Resistance

**DOI:** 10.3390/biomedicines9111512

**Published:** 2021-10-21

**Authors:** Desh Deepak Singh, Amna Parveen, Dharmendra Kumar Yadav

**Affiliations:** 1 Amity Institute of Biotechnology, Amity University Rajasthan, Jaipur 303002, India; ddsbms@gmail.com; 2 College of Pharmacy, Gachon University of Medicine and Science, Hambakmoeiro 191, Yeonsu-gu, Incheon 21924, Korea

**Keywords:** breast cancer, PARP (poly(ADP-ribose) polymerase), TNBC, therapeutic target, DNA damage repair, signaling pathway

## Abstract

Triple-negative breast cancer is a combative cancer type with a highly inflated histological grade that leads to poor theragnostic value. Gene, protein, and receptor-specific targets have shown effective clinical outcomes in patients with TNBC. Cells are frequently exposed to DNA-damaging agents. DNA damage is repaired by multiple pathways; accumulations of mutations occur due to damage to one or more pathways and lead to alterations in normal cellular mechanisms, which lead to development of tumors. Advances in target-specific cancer therapies have shown significant momentum; most treatment options cause off-target toxicity and side effects on healthy tissues. PARP (poly(ADP-ribose) polymerase) is a major protein and is involved in DNA repair pathways, base excision repair (BER) mechanisms, homologous recombination (HR), and nonhomologous end-joining (NEJ) deficiency-based repair mechanisms. DNA damage repair deficits cause an increased risk of tumor formation. Inhibitors of PARP favorably kill cancer cells in *BRCA*-mutations. For a few years, PARPi has shown promising activity as a chemotherapeutic agent in *BRCA1*- or *BRCA2*-associated breast cancers, and in combination with chemotherapy in triple-negative breast cancer. This review covers the current results of clinical trials testing and future directions for the field of PARP inhibitor development.

## 1. Introduction

**Figure 1:**
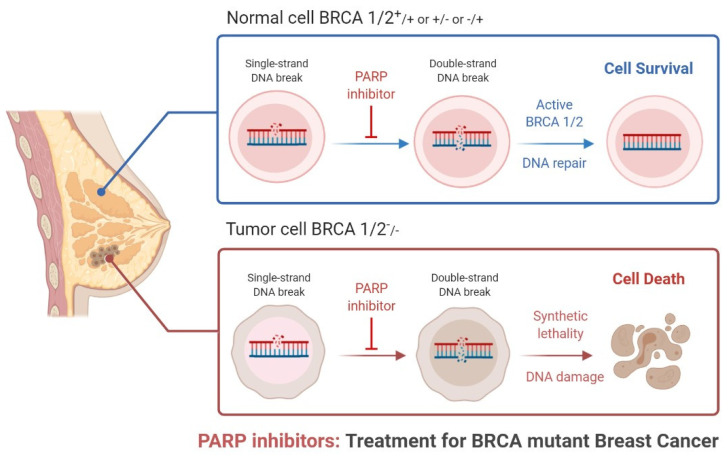
Role of PARP inhibitors in treatments for *BRCA* mutant breast cancer.

**Figure 2:**
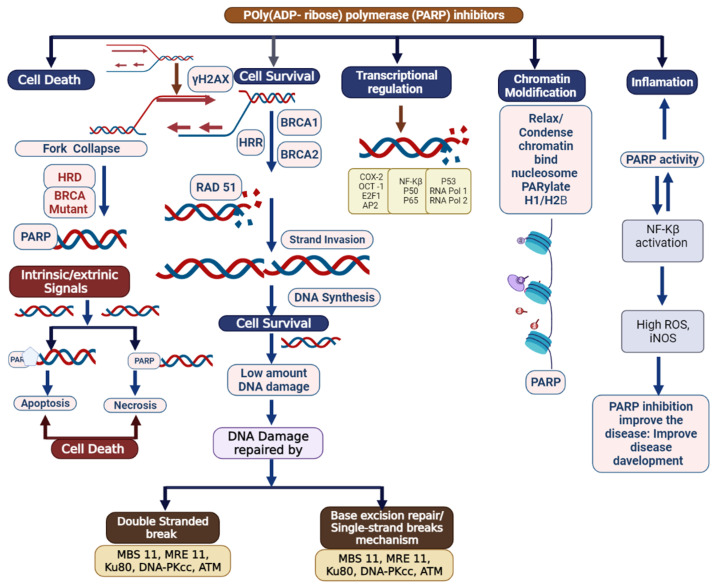
Schematic delineating the multifaceted nature of poly (ADP) ribose polymerase (PARP): DNA repair, chromatin modification, inflammation, transcriptional regulation, and cell death. Potential role of elevated PARP-1 in tumorigenesis. After DNA damage, PARP-1 activates DNA repair. However, PARP-1 also acts as a co-activator of NFkB signaling, which can propagate inflammatory signaling and lead to more DNA damage, including the formation of oxidatively clustered DNA lesions (OCDLs). The formation of OCDLs is elevated in numerous tumor types. PARP-1 activity could potentially be beneficial or harmful in the repair of ROS-induced DNA lesions.

Breast cancer (BC) is the most common cancer that occurs in women worldwide [1]. BC is caused by accumulation of somatic mutations in breast cells, which impair cell division and DNA repair mechanisms, resulting in irregular cell growth proliferation, differentiation, and ultimately, progression of tumorigenesis [2,3]. Triple-negative breast cancer is more belligerent and has a poorer prognosis than other types of breast cancer. Triple-negative breast cancer (TNBC) accounts for approximately 15% of all BC, and lacks human epidermal growth factor receptor 2 (HER2), progesterone receptor (PR), and estrogen receptor (ER) expression and amplification. If we compare it with another type of BC, TNBC exhibits inherently aggressive clinical symptoms and poorer clinical outcomes [4,5,6,7]. Presently, the clinical targeted drugs for BC include poly-(ADP)-ribose polymerase (PARP) inhibitors (PARPi), CDK4/6 inhibitors (CDK4/6i), PI3K inhibitors, and AKT inhibitors—but none of these drugs alone are very effective against TNBC [8]. There is an urgent need for the rational exploration of drug compatibility and potential targets for TNBC [7,8]. PARP1 (poly (ADP-ribose) polymerase 1) was discovered approximately 50 years ago and is involved in gene transcription, DNA repair, and cell death [9]. PARP1 has acceptable therapeutic importance against cancer, as shown in Figure 1 and Figure 2 [10]. PARP inhibitors have emerged as effective treatments in clinical trials for sporadic TNBC and *BRCA*-associated cancers [11]. There are various types of PARP inhibitors under clinical trial such as olaparib, BSI-201, talazoparib, rucaparib veliparib, and niraparib [10,11]. Inhibition of the PARP-1 and PARP-2 enzymes is believed to be attained mainly via binding of the NAD+ catalytic domain side chain, extending out of the NAD catalytic site of the PARP inhibitor [12]. It also thought that the PARP enzyme locks on to the site of DNA damage, preventing its usual release from DNA molecules [10,11,12,13,14,15]. PARP-1 binds to the damaged site through its zinc-finger domains in the presence of SS (single-stranded)-DNA breaks [13]. PARP-1 and poly (ADP) polymerization recruits and binds other DNA-repair proteins, leading under normal cell physiology to an increasingly negative charge on the enzyme, and eventual dissociation from the DNA [14]. Some clinical investigations have shown the need for HRD (homologous recombination DNA repair) in facilitating PARP inhibition, via loss of *BRCA* function [16,17]. Researchers from the field have suggested that PARP inhibition is associated with the induction of DNA damage by chemotherapy in the more general cohort of TNBC.

## 2. Mechanism of Poly (ADP-Ribose) Polymerase Inhibition

**Figure 3:**
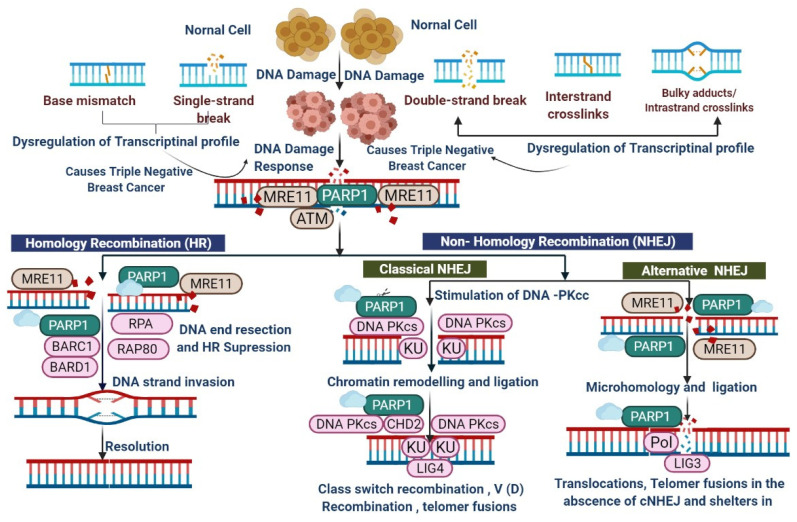
PARP1 is required for the robust detection of DNA double-strand breaks (DSBs) and for the initial DNA damage response through its interaction with MRE11 (meiotic recombination 11) and the apical checkpoint kinase ATM (ataxia telangiectasia mutated). PARP1 has a role in DNA end resection during the HR process through the recruitment of *MRE11* to DSBs, which is followed by binding of the single strand by replication protein A (RPA); the reaction is carried out with *BRCA1*-associated *BARD1* (RING domain protein 1). PARP1 also actively participates in DSB repair by activating NHEJ. When DSBs are directed for repair by cNHEJ, they are mediated by KU70KU80 dimers, which trigger DNA-PKcs (DNA-dependent protein kinase catalytic subunits). PARP1 interacts with DNA-PKcs and activates them without the requirement of KU proteins. PARP1 also plays an important role in chromatin remodeling during cNHEJ by facilitating the recruitment of *CHD2* (chromodomain helicase DNA-binding protein 2), which then activates XRCC4 (X-ray repair cross-complementing protein 4) and LIG4 (DNA ligase 4) for DNA ligation. PARP1 has a role in alternative NHEJ (aNHEJ), which is active in the absence of cNHEJ. aNHEJ requires the processing of DNA ends by *MRE11*, which is recruited by PARP1. The resected ends are then joined through sequence microhomology, and the gap is filled by Pol theta (DNA polymerase θ) and ligated by LIG3.

**Figure 4:**
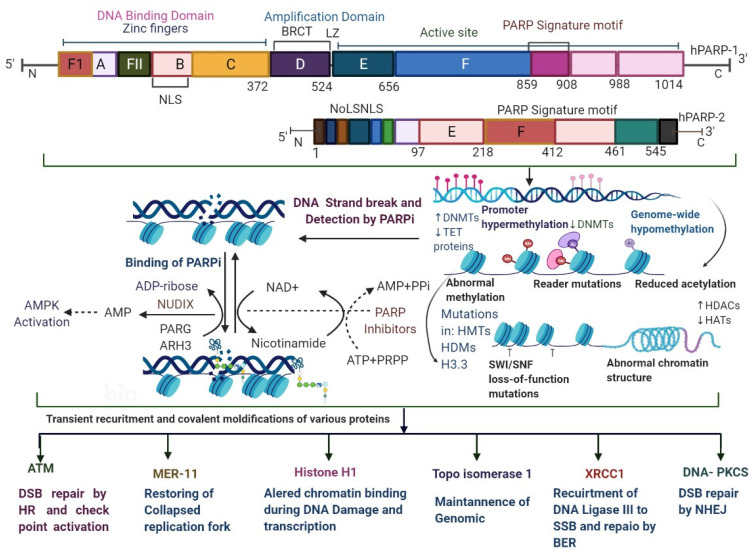
The biochemical functions of PARP1 in DNA damage repair. The domains of PARP1 are shown; PARP1 has a DBD, which contains ZF13 (zinc finger motifs) and NLS (nuclear localization signals); its central domain contains the interaction motifs *BRCA1* C-terminal domain and a carboxy-terminal catalytic domain, which contains a signature of PARP and CD. The CD contains the active site and binds to NAD+, as well as to the Trp–Gly–Arg (WGR) domain. PARP1 detects DNA damage through its DBD, and it is activated by synthesizing PAR (poly(ADP-ribose)) chains—mainly on itself, but also on some of its target proteins. NAD+ is used as a substrate for PAR formation and is replenished in cells from nicotinamide using ATP. PAR chains are rapidly catabolized by *PARG* (PAR glycohydrolase), *ARH3* (ADP-ribosylhydrolase 3), and *OARD1* (O-acyl-ADP-ribose deacetylase 1). *PARG* cleaves the glycosidic ribose–ribose bonds of PAR. Histone deacetylase genes (HDACs) increase the expression of genes through transcription activation By deacetylating the histone tails, the DNA becomes more tightly wrapped around the histone cores, making it harder for transcription factors to bind to the DNA, which could be attributed to decrease in histone acetyltransferase (HAT). However, cleavage of the terminal ADP-ribose moiety requires *OARD1*, and results in the release of mono(ADP-ribose). Poly(ADP) ribosylation (PARylation) of PARP1 and other target proteins, both covalently and non-covalently, results in the recruitment of multiple proteins that have roles in different aspects of DNA damage repair.

PARP1 is associated with a superfamily of ADP-ribosylating enzymes (ADPRE); it acts as a catalyst for the transfer of ADP-ribose residues from NAD+ onto target substrates, constructing a chain of poly((ADP-ribose); PAR) [17,18]. The formation of PAR chains occurs commonly in eukaryotic cells [17]. ADPRE consists of the PARP1 homology protein, which is also produced by the catalytic activity of ADP-ribosyl transferase reactions [19]. The PARP family has approximately 17 members; a few of them (PARP1, PARP2, PARP5A, and PARP5B) are synthesized 1–3PAR chains [11]. Usually, enzymes from this family produce single ADP-ribose units called MARs (mono (ADP-ribosyl)ases) [18,19]. A researcher in this field has found that PARP1 is actively involved in DNA repair and has shown an association of it with nuclear enzymes and chromatin, as shown in Figure 2, Figure 3 and Figure 4 [20].

The role of PARP1 in DNA repair mechanisms was assumed in 1975, after which, more effective PARP inhibitors were established in 1990, which were found to be more effective in DNA repair mechanisms, as shown in Figure 2, Figure 3 and Figure 4 [12,13,14,15,16,17]; these are triggered by topoisomerase 1 toxicity and prevent re-ligation of DNA at SSBs [20]. PARP1 distinguishes the SSB via its DNA-binding domain with motifs of three zinc fingers, which cause a change in their conformational structure and trigger PARP1 to cleave NAD+, as shown in Figure 4 [21,22,23,24]. The ADP-ribose moiety covalently attaches to either PARP1 or other nuclear proteins, such as histones; other ADP-ribose groups are then added to it to produce long and sometimes branching PAR chains [22,23]. SSBs are identified and repair DNA damage by DSB (DNA double-strand break) repair pathways via the homologous recombination (HR) and non-homologous end-joining (NHEJ) pathways. Damaged DNA at broken chromatin terminals is repaired by alt-NHEJ (alternative end-joining) and MMEJ (microhomology-mediated end-joining) [23,24,25,26]. HR acts as a conventional repair mechanism with reliability in the S and G2 phases of the cell cycle [27,28]. *BRCA1* plays an important role in cell cycle regulation and SSB; *BRCA2* mediates the important recombination enzyme *RAD51* and regulates DNA repair mechanisms, as shown in Figure 3 [29,30]. *BRCA1* mutations are linked to TNBC and *BRCA2* mutations are associated with ER/PR-positive tumors [3,16]. DNA damage repair is associated with the HRR pathway and accomplished by *MRE11*, *RAD50*, and Exo1 (MRN complex). PARP trapping occurs via the NHEJ pathway at the replication fork via enzymes such as DNA-PK, *XRCC4*, and Ku70/80, which are employed to undertake the DNA repair process [31,32,33]. Another pathway of MMEJ or NHEJ leads to chromosomal and genomic instability and causes tumor cell death or somatic mutations, as shown in Figure 3 [31]. Treating TNBC by PARPi is an important mechanism due to HR deficiency and accumulated mutations. In response to DNA damage, PARylation occurs [31,34], and RNAi-mediated depletion of PARP1 encourages the destruction of *BRCA1/2*-lacking tumor cells [16]. PARPi treatment displays antitumor effects in patients with *BRCA1/2* mutations [3,16]. Approximately 10–20% mutations have been observed in TNBC patients [2,3]. PARPi have shown effective clinical outcomes (Table 1 and Table 2) [34,35,36,37]. The FDA (Food and Drug Administration) has approved olaparib and talazoparib for the treatment of TNBC patients with *BRCA1/2* mutations (Table 1, Table 2 and Table 3) [16,35,36,37,38,39]. Researchers from the field have reported that HR mechanisms also repair genetic mutations other than those in *BRCA* genes, such as *CDK12*, *RAD51B*, *RAD51C*, *RAD51D*, *CHEK1*, *CHEK2*, *FANCA*, *FANCC*, * FANCD2*, * FRANCE*, * FANCF*, * ATM*, * PALB2*, * NBS1*, * ATR*, * BAP1*, * WRN*, *MRE11A*, and *BLM* [3,31,32,33,34,35,36,37,38,39,40]. Similar treatment responses in cancers with *BRCA*ness and *BRCA1/2*-mutated tumors have been observed [3,16]. PARPi limits the DNA-damage response induced by chemotherapy and radiotherapy (Table 1, Table 2 and Table 3) [40].

### 2.1. PARP Inhibition and the PI3K/AKT/mTOR Pathway

TNBCs exhibit aberrant initiation of the PI3K pathway via various mechanisms; the PI3K/AKT/mTOR pathway has been investigated for therapeutic strategies in patients with TNBC [3]. PI3K is also actively involved in the DSB repair mechanism via use of the HR complex [41]. Researchers from the field have investigated the inhibition of *PI3K*, which causes DNA damage, downregulates *BRCA 1* and *2*, increases poly-ADP ribosylation and, finally, activates PARP inhibition. *PI3K* and PARP inhibitors have been investigated in a mouse model for *BRCA1*-related tumors, and provide synergistic effects in their treatment [42]. In addition, *PI3K*-mTOR is inhibited by GDC-0980, and was tested in combination with veliparib and carboplatin in a TNBC model. Furthermore, a combination of veliparib and carboplatin has been shown to be effective against xenograft tumors [40]. The mTOR signaling pathway is an important target strategy for TNBC [42]—it causes downregulation of the *PI3K* pathway and also suppresses the regulation of TNBC cell lines [3,42]. A combination of PARP inhibitors and mTOR inhibitors has shown significant activity in TNBC cell lines [43,44].

### 2.2. PARP Inhibitors Combined with CDK1 Inhibitors

*CDK1* is required for HR and check-point activation and is mediated by *BRCA1* [43]. *CDK1* inhibition modulates the repair of DNA by HR [43,44]; *PARP* and *CDK1* inhibition in *BRCA* reduces the formation of tumors in xenograft models. *CDK1* and *PARP* inhibition has been shown to be a promising emerging approach for *BRCA* tumor treatment [45,46]. *CDK1* activity is impaired by *BRCA1*, working via the HR repair mechanism [46]. Researchers from the field have investigated the combined effects of *CDK1* inhibition using RO3306 and olaparib in *BRCA* tumors [29].

### 2.3. PARP Inhibitors Combined with Histone Deacetylase Inhibitors

HDI (histone deacetylase inhibitors) stimulate tumor cells to inhibit PARP and enhance antitumor activity against TNBC cells based on their *BRCA*ness [47]. *HAD* blockade prevents deacetylation of HSP90 (heat shock protein 90) and leads to inhibition of hyperacetylation; as a result, HSP70 cannot interact with *CHK1*, *RAD52*, *ATR*, and *BRCA1* [48,49]. HDI and PARP inhibitors are considered epigenetic drugs [47,48]. The combined drugs act synergistically; combinations of HDI and PARP inhibitors have been clinically evaluated for treatment of neoplastic diseases [50]. Cell proliferation inhibition is linked with rising apoptosis levels, leads to DNA damage, and alters the cAMP signaling mechanism [10,11,12,13,14,15]. The combined effects of SAHA and PJ34 have shown effective results in leukemia cell lines [51]. Combinations of *HDI* and PARP inhibitors can reduce proliferation and induce apoptosis. Additionally, they are emerging as an effective treatment approach for TNBC [47,50,51].

### 2.4. PARP Inhibitors Combined with EGFR Inhibitors

In TNBC patients, overexpression of EGFR is observed and associated with a basal-like phenotype [3]. EGFR is a transmembrane receptor that stimulates growth factor signaling pathways, such as the HER1 family, and is actively involved in cell cycle regulation, differentiation, proliferation, and survival [52,53]. EGFR-targeted therapies are based on tyrosine kinase inhibitors (TKIs), monoclonal antibodies, and combination chemotherapy [53]. EGFR inhibition changes the DNA DSB repair capacity of treated cells; EGFR1 and 2 inhibitor lapatinib encourages a momentary DNA repair discrepancy in human TNBC cells and then enhances the cytotoxicity of the PARP inhibitor veliparib [40]. The targeting of MET, which acts as a regulator for EGFR tyrosine kinase phosphorylation, has shown effective results in patients with TNBC in combination with EGFR inhibition [40,52,53].

### 2.5. ATM Downregulation and PARP Inhibition

Ataxia-telangiectasia mutated (ATM) is a tumor suppressor gene whose stimulation is linked to oxidative stress and which can moderate the cellular response to oxidative stress [3,54,55]. ATM-based regulation of pexophagy, proteostasis, mitophagy, and autophagy highlights the need to remain aware that the consequences of ATM expression and signaling pathways are dependent on the specific cellular context. Investigations are urgently needed to determine the molecular mechanisms of the cytosolic functions of ATM that could modify tumor development and therapy [54,55]. Researchers from the field have used genetic modification to suppress ATM expression, using iniparib and olaparib with PARP inhibitors [29,56]. After treatment with iniparib and olaparib, different types of responses are produced [56]. Olaparib treatment was more effective than iniparib against TNBC [29]. Transforming growth factor-beta (TGF-β) suppresses ATM in BC cells by stimulating the miR-181 family and targeting the 3′ untranslated regions of ATM transcripts [57]. TGF-β activates this PARP inhibitor mechanism, as demonstrated in preclinical in vitro and in vivo TNBC models [3,41,57].

### 2.6. PARP Inhibitors Combined with Androgen Receptor (AR) Inhibitors

AR is expressed in 15%–50% of TNBCs and represents an opportunity for targeted therapy of TNBCs [58]. A combination of AR inhibitors and PARP1 inhibitors could be a promising target for sporadic positive-AR expression and methylation-mediated *BRCA1* dysfunction in patients with TNBC [58,59]. The AR antagonist MDV3100 (enzalutamide) has an antitumor potency greater than first-generation AR inhibitors; it suppresses AR nuclear translocation and decreases DNA binding and coactivator recruitment [60]. Researchers from the field have reported effective responses against MDV3100 in AR-positive BT-549 cells and resistant responses in AR-negative MDA-MB-468 and MDA-MB-231 cells [61]; MDV3100 improved the PARP1 inhibitor olaparib-mediated reduction of cell viability in AR-positive and *BRCA1*-inactivated BT-549 cells in vitro and in vivo. AR antagonism with PARP1 inhibitors can be an effective target in patients with TNBC [62,63]. Enzalutamide with olaparib inhibited proliferation and suppressed the growth of prostate cancer xenografts in mice [64]. Given that one of the TNBC subtypes (LAR) expresses androgen receptors, the combination of antiandrogens and PARP inhibitors could be an effective treatment for this subset of patients with TNBC [3,62,64].

## 3. Clinical Applications of PARP Inhibitors in TNBC

PARP inhibitors have been shown to have effective clinical outcomes against different types of cancer. There are various clinical trials registered investigating PARPi therapies (Table 1, Table 2 and Table 3).

### 3.1. Olaparib

Olaparib is a potent oral PARP inhibitor effective against *BRCA1* and *BRCA2* mutations [29]. A multicentric clinical evaluation of olaparib was carried out using it as a monotherapy for inpatients with germline *BRCA1/2* mutations [56,62,63]. Olaparib was administered to the patient twice a day at a dose of 400 mg. The clinical trial was performed with 298 patients, out of which, effective clinical therapy was observed in 12.9%, with adverse effects of vomiting, nausea, and fatigue observed [64,65,66,67,68]. Another study was performed to optimize the drug concentration and determine its maximum dose and minimum dose. In patients with *BRCA1* and *BRCA2* mutations, an ORR (overall response rate) of 11 (41%) was observed in 27 patients with a 400 mg dose twice daily. An ORR of 6 (22%) was observed in 27 patients with 100 mg doses twice daily; the ORR was observed to be 7/13 (54%) with higher doses and 4/16 (25%) with lower doses in TNBC patients. In higher dose-tested patients, some adverse effects were observed, such as anemia, vomiting, nausea, and fatigue. Olaparib was approved by the FDA based on the clinical outcomes of the patient [65,66,67,68]. A phase 3 clinical trial employed olaparib monotherapy in germline *BRCA* mutations with *HER2* negativity and, at minimum, previous chemotherapy therapy [67]. A total of 300 patients were selected randomly in a 2:1 ratio into two groups; group one was administered 300 mg olaparib twice daily, and 92 patients in group two were administered vinorelbine or capecitabine and eribulin in 21-day cycles. Out of 300, 49.8% of the TNBC patients were included in the olaparib group and 49.5% of the TNBC patients received standard therapy [3,64,65,66]. Median PFS was significantly longer in the olaparib group than in the standard therapy group (7.0 months vs. 4.2 months; hazard ratio for disease progression or death, 0.58; 95% CI, 0.43 to 0.80; *p* < 0.001). In the subgroup analysis, the hazard ratio for PFS was 0.43 (95% CI, 0.29–0.63) for patients with TNBC [3,66,67,68]. The response rate was 59.9% in the olaparib group and 28.8% in the standard therapy group, while the rate of grade 3 or higher adverse events was 36.6% in the olaparib group and 50.5% in the standard therapy group; the rate of treatment discontinuation due to toxic effects was 4.9% and 7.7%, respectively [66,67,68,69]. Metabolism of olaparib occurs via oxidation and dehydrogenation and does so progressively via the use of other factors such as sulfate conjugate and glucuronide [65,66]. Olaparib is mainly excreted through urine (44%) and feces (42%) [66].

OlympiAD was a randomized open clinical phase III trial (NCT02000622) assessing the daily administration of 600 mg olaparib tablets. A total of 302 patients who had received two or fewer prior treatments were randomized in a 2:1 ratio to olaparib or chemotherapy. The results showed significantly prolonged PFS with olaparib versus standard therapy (7.0 vs. 4.2 months; hazard ratio (HR), 0.58; 95% CI, 0.43–0.8; *p* < 0.001); Response rates were observed to be 59.9% vs. 28.8% (olaparib vs. standard group) [64]. Olaparib was the first PARP inhibitor to establish higher efficacy and tolerability than standard chemotherapy in g*BRCA*-mutated advanced BC [65,66,67,68]. According to earlier results, the FDA approved olaparib as the first PARP inhibitor for the treatment of this patient subgroup. However, in the interim analysis, no differences in overall survival (OS) were observed between the two groups [68,69,70]. The 3-year OS was 40.8% versus 12.8% in the two groups, respectively, in patients with TNBC. Currently, research on PARP inhibitors for adjuvant therapy and neoadjuvant therapy, as well as for the prevention of BC, is ongoing—including the OlympiA (phase III) and GeparSixto studies; in the future, the results of these studies will evaluate adjuvant therapy with olaparib for *HER-2*-/g*BRCA*m BC and explore the value of a PARP inhibitor in neoadjuvant therapy, respectively [71,72,73]. Various remarkable drugs have been approved to benefit patients with TNBC, including the PARP inhibitors olaparib and talazoparib for germline *BRCA* mutation-associated breast cancer (g*BRCA*m-BC) and immunotherapy using the checkpoint inhibitor atezolizumab, in combination with nab-paclitaxel for programmed cell death-ligand 1-positive (PD-L1+) advanced TNBC [66,73].

### 3.2. Iniparib (BSI-201)

Iniparib was the first potent PARP1 inhibitor, effective against cancer cell lines with 40–128 μM IC50 values, and is not toxic at 200 mg/kg in Syrian hamsters [74,75]. The efficacy of iniparib (BSI-201) was established by caspase-3 and TUNEL staining of OVCAR-3 tumors; iniparib efficacy was high in combination with topotecan [70,71,72]. Iniparib used together with a PARP-1 inhibitor has also shown efficacy in DNA repair mechanisms [72]. One clinical trial investigated patients with metastatic TNBC [74,75], in which a total of 123 patients were selected randomly and two groups were made; in each group, patients received 1000 mg/m^2^ gemcitabine and carboplatin on days 1 and 8, either with or without 5.6 mg/kg iniparib on days 1, 4, 8, and 11, over a cycle of 21 days [74,76]. The clinical efficacy of iniparib was increased with carboplatin and gemcitabine, and the ORR was increased from 32% to 52%. The time duration of the iniparib dose was also increased from a median PFS of 3.6 months to 5.9 months, and the median ORR from 7.7 months to 12.3 months; the hazard ratio for death was observed to be 0.57; *p* = 0.01. ORR and PFS were analyzed further in a phase III clinical trial; the trial did not find successful treatment of patients [76].

### 3.3. Niraparib

Niraparib is a PARP1 and PARP1 inhibitor. Niraparib is indicated as a maintenance treatment for recurrent cancer patients, mainly with HR deficiency (HRD) with positive status [73,77]. HRD has been linked to deleterious *BRCA* mutations in patients, with disease development occurring more than six months later following platinum-based chemotherapy [73,77]. Niraparib was extended for use in the care treatment of adults following first-line platinum-based chemotherapy [67,73].

Patients with solid tumors (*BRCA1* or *BRCA2* mutation carrier) were enrolled in a phase I clinical trial [76,77,78,79]. The currently used therapeutic option was tested along with niraparib in *BRCA*-mutated metastatic breast cancer; patients with germline *BRCA* mutations were treated with a PARP inhibitor rather than chemotherapy, and the availability of PARP inhibitors increased [76,77,78,79]. No safety concerns have been noted by the IDMC (Independent Data Monitoring Committee) concerning niraparib [76,77,78,79]. The clinical outcome from the BRAVO (Breast Cancer Risk and Various Outcomes) trial is expected to be supportive of a planned trial of niraparib in combination with an anti-PD-1 antibody in women with metastatic TNBC [76,77,78,79].

### 3.4. Veliparib (ABT-888)

Veliparib (ABT-888) is a potent PARP1 and PARP2 inhibitor used as a neoadjuvant. It has good pharmacokinetic properties and has shown effective clinical outcomes [80]. Veliparib is effective in platinum-based therapy in xenograft models [80,81]. Significantly, the eradication of solid tumors following neoadjuvant chemotherapy, designated the clinical–pathological response in breast and axillary nodes during surgery, is connected with progression-free survival (PFS) and overall survival rates (OSRs)—with strong correlations in TNBC and *HER2*-positive disease, raising interest in the neoadjuvant approach [82,83]. Veliparib was clinically evaluated in TNBC patients in combination with carboplatin; it was also tested against the NAD+ catalytic enzyme SIRT2, showing inactivity against >5000 nM of the enzyme. Receptor-binding assays were performed in 74 patients for Veliparib receptor profile analysis at a concentration of 10 μM [81,82,83]. Multiple investigations were carried out, such as control-specific binding at 50% of human 5-HT7 (84%) sites with an IC50s value of 1.2 μM; IC50s at H1(61%), with an IC value of 5.3 μM; and human 5-HT1A (91%) with a IC50s value of 1.5 μM. c-Met knockdown cells show shMet-A (95% CI = 4–4.5) tumor growth retardation with up to 60 μM Veliparib (ABT-888) [81,82,83]. When treated with 38 μM Veliparib, c-Met knockdown cells show shMet-B (95% CI = 1.3–2.5) tumor growth inhibition. Cell viability was higher with 1,000 µM sulfur mustard (SM) exposure in HaCaT cells at 6 h post-treatment by Veliparib [81,82,83]. Additionally, Veliparib no longer shows protective effects at 24 h post SM exposure.

Randomized patients were selected to receive either paclitaxel as monotherapy or veliparib and carboplatin as a combination therapy, followed by doxorubicin and cyclophosphamide given in four cycles [83]. Clinical outcomes were examined, with estimated rates of PCR of 51% in the combination group with TNBC patients and 26% in the control group of patients [83]. For the phase III clinical trial, 634 patients were selected based on histological clinical stage II–III TNBC with no previous therapy for potentially curative surgery—they were randomly assigned to two groups; group I was treated with 50 mg veliparib orally twice a day, with 12 weekly doses of 80 mg/m^2^ intravenous paclitaxel, and carboplatin administered every 3 weeks, for 4 cycles [81,82,83]. Patients with a germline *BRCA* mutation were then allocated to group II and administered cyclophosphamide and doxorubicin every 2–3 weeks for 4 rounds [82]. Effective clinical outcomes were observed to be higher in 53% of patients with combined therapies in comparison to patients who received paclitaxel alone (31%). No significant toxicity was observed against Veliparib. [81,82,83].

### 3.5. Talazoparib (BMN-673)

Talazoparib is a PARP inhibitor that is hypothesized to have a higher effectiveness than olaparib due to the process of PARP trapping, in which a PARP molecule is trapped on the DNA, inhibiting cell division [84]. Talazoparib is a dual-mechanism PARP inhibitor that traps PARP on DNA [84,85]. The phase II study ABRAZO evaluated the efficacy of talazoparib on inpatients with germline *BRCA1/2* mutations before being treated with platinum or multiple regimens [84,85]. Clinical efficacy was evaluated in TNBC/HR+ patients at 26%/29%; adverse effects were observed such as neutropenia, thrombocytopenia, anemia, fatigue, nausea, and diarrhea [85]. A phase III clinical trial was performed to compare the efficacy and safety of talazoparib in TNBC patients [84,85,86]. Clinical efficacy was observed—median PFS was 8.6 months for talazoparib, with a 46% reduction in the tumor, and 5.6 months for chemotherapies such as capecitabine, eribulin, gemcitabine, or vinorelbine [84,85,86]. All key secondary efficacy endpoints (OS, ORR, clinical benefit rate at 24 weeks) demonstrated benefits with talazoparib [85,86]. The PARP inhibitor was generally well tolerated, with minimal non-hematologic toxicity and few adverse events associated with treatment discontinuations [84,85,86]. Patients were treated with an anthracycline, with or without taxane as a neoadjuvant [84]; its primary clinical efficacy was examined, with PFS performed according to RECIST 1.1 criteria: median PFS was 8.6 and 5.6 months in the talazoparib and chemotherapy arms, respectively (HR 0.54; 95% CI: 0.41, 0.71; *p* < 0.0001) [85,86]. Its clinical approval was considered in EMBRACA (NCT01945775), an open label trial randomizing 431 patients (2:1) who were g*BRCA*m *HER2*-negative to treatment with talazoparib (1 mg) with no more than 3 prior cytotoxic chemotherapy treatments for metastatic disease. Talazoparib was approved by the FDA for germline *BRCA*-mutated (gBRCAm), *HER2*-negative locally advanced or metastatic breast cancer. The FDA also approved the BRAC Analysis CDx test for identifying patients with breast cancer with deleterious or suspected deleterious g*BRCA*m who are eligible for talazoparib [85].

### 3.6. Rucaparib

Rucaparib is an effective inhibitor of PARP1, PARP-2, and PARP-3 in *BRCA*-mutated patients (germline and/or somatic). Rucaparib was also found to be effective in HR-deficient patients [87]. Rucaparib is indicated as a monotherapy treatment for adults who are platinum-sensitive, patients who have been treated with two or more prior lines of platinum-based chemotherapy, and for those who are unable to tolerate further platinum-based chemotherapy [88]. A multicenter phase clinical trial was performed to establish *BRCA1/2* mutations and earlier treatment with rucaparib. Intravenous, and subsequently oral, rucaparib were evaluated with different dose concentrations [89]. Efficacy and safety levels were evaluated, such as pharmacodynamics, pharmacokinetic dose-limiting toxic effects, and tolerability [90]. Intravenous rucaparib was given and the objective response rate was analyzed: 41% of patients showed an ongoing response for at least 12 weeks [91]. The efficacy and safety of rucaparib in patients with *HER2*-negative metastatic breast cancer were associated with *BRCA*ness phenotype and/or a somatic *BRCA* mutations [87,88,89,90,91]. Patients received 600 mg rucaparib orally for 21 days or up to the development of the disease. The main endpoint was the clinical benefit rate and secondary endpoints, including PFS, overall survival, safety, and the prognostic value of the *BRCA*ness signature [87,88,89,90,91]. An additional study determined the quantity of sporadic TNBC patients likely to benefit from rucaparib treatment [87,88,89,90,91].

### 3.7. Checkpoint Inhibitors

TNBC is pushing to improve treatment by answering questions regarding biomarkers of response, defining the utility of neoadjuvant approaches, and exploring potential combinations of checkpoint inhibitors and PARP inhibitors. The FDA approved the nab-paclitaxel (Abraxane) with atezolizumab (Tecentriq) for patients with metastatic PD-L1-positive TNBC. The approval was based on the phase 3 IMpassion130 trial (NCT02425891), which established a 38% decrease in the risk of disease development with the combination vs. placebo plus nab-paclitaxel in this patient population. Pembrolizumab is a second approved checkpoint inhibitor drug, approved by the FDA in Nov 2020, for patients with metastatic TNBC whose tumors express a PD-L1 combined positive score (CPS) of 10 or higher, as determined by an FDA-approved test. Pembrolizumab also demonstrated proof of concept as a neoadjuvant based on findings from the phase II I-SPY2 trial (NCT01042379); pembrolizumab neoadjuvant plus chemotherapy extended pathologic complete response (pCR) rates by 13.6 percentage points compared with chemotherapy alone for patients with early TNBC (95% CI, 5.4–21.8; *p* < 0.001).

## 4. PARP Inhibitor Resistance

*BRCA1* and *2*-deficient cancer cells are sensitive to PARP inhibitors (PARPi); various PAPRi have been permitted for treatment of BC [53,89,90]. Nevertheless, PARPi resistance has been observed during patient treatment. Most *BRCA1/2*-deficient patients fail to respond to PARPi with prolonged treatment [90]. In PARPi, HRD plays an essential role in preventing the growth of tumor cells. Researchers from the field have reported that DNA replication fork protectors are also involved in PARPi resistance in *BRCA1/2*-deficient patients [89,90,91]. Therefore, various factors are responsible for resistance, such as epigenetic modification, restoration of ADP-ribosylation, pharmacological alteration, and reversion mutations.

### 4.1. Restoration of HR Repair in PARPi Resistance

HR dominates in the S/G2 cell cycle due to the high DNA replication [92]. The DSB ends are firstly resected by the *MRN* (*Mre11–Rad50–Nbs1*) complex with *EXO1*, DNA2, and *MUS8*, leading to the development of the ssDNA and pushing the cells towards HR [92,93]. Subsequently, the resected DNA ends are coated by the hyperphosphorylated single-stranded DNA binding protein A (RPA) [94,95]. The variant *H2AX* is stimulated and phosphorylated by apical kinases, such as ATM and ATR (ATM and Rad3-related). The distribution of γ*H2AX* on the chromosome promotes the accumulation of additional DDR proteins, including 53BP1 (p53-binding protein) and *BRCA1* to the *DDR* foci [55,95]. With the help of *PALB2*, *BRCA2* binds with *BRCA1* and promotes the loading of recombinase *RAD51* on the ssDNA [95]. Hence, the repair of the HR pathway via encouragement of DNA end resection and development of nucleoprotein filaments and D-loops can promote PARPi resistance [92,93,94,95].

### 4.2. DNA end Resection in PARPi Resistance

CDK12 deletion is responsible for PARPi resistance in TNBC patients; CDKs prevent DNA end resection and lead to PARPi resistance [96]. Most PARPi and CDK inhibitors are used by clinicians [97]. In DNA end resection, PARPi resistance may occur due to some important factors of the cell cycle, as well as CDKs, such as *RIF1*, *REV7*, and *53BP1* [98]. Chromatin-binding protein (53BP1) plays a very important role in blocking DNA resectioning by controlling CtIP transport to DSB sites, leading to PARPi resistance [99]. *53BP1* is responsible for DNA ends shielding in two pathways: firstly, by supporting the nucleosomal blockade of end-resection nucleases by H4K20m2 and H2AK15ub. In the second pathway, the effector protein complex is responsible for protecting DNA ends such as *REV7*, *SHLD1*, *SHLD2*, and *SHLD3* with the help of 53BP1 and RIF1 [98,99]. NHEJ repair and HR repair mechanisms are also linked with PARPi resistance, with *REV7* mainly responsible for sensitizing cells to PARPi. Conformational changes occur in *REV7*, linked with TRIP13 ATPase-promoted HR—leading to PARPi resistance [11,96]. The protective role of *53BP1* has required the interaction with *PTIP* and *RIF1*, which is dependent on ATM [98,99]. Therefore, the combined interaction of *53BP1* and *RIF1* is involved in DNA end resection and PARPi resistance.

### 4.3. Formation of RAD51-ssDNA Filament and D-Loop in PARPi Resistance

RAD51-ssDNA plays an important role in HR repair, and RAD51 acts as a biomarker for HR repair and PAPRi resistance in patients with *BRCA* mutations [48,92,96]. *EMI1* has been recognized as an essential target of *RAD51* and modulator of PARPi activity [48,92]. PARPi resistance in *BRCA1*-deficiency occurs in TNBC cells due to downregulation of EMI, leading to accumulation of RAD51 [96]. In TNBC cells, *RAD51* degradation occurs due to DDB2 (damaged DNA binding protein) and DNA damage recognition factor [48,96]. Defective HR and sensitivity to PARPi inhibit DDB2-induced *RAD51* polyubiquitination. *RAD51* phosphorylation takes place in the presence of *TOPBP1* (topoisomerase IIβ-binding protein) [48,92,96]. Another protein, *BRD4* (bromodomain protein 4) is responsible for genome stability; the inhibition of *BRD4* causes the aggregation of *RAD51*, lacking stimulation by the ATR-dependent/ATM DNA damage response [96,100,101]. DBRD4 inhibitors (JQ1, INCB054329) increase the activity of PARPi. APRIN and PALB2 to favorably bind to D-loop constructions and directly interact with *RAD51* to activate HR. It has been shown that deletion of APRIN and PALB2 stimulates BRCAness and directs PARPi to cells [100,101]. Furthermore, Pol δ assists in D-loop development and amplifies the activity of HR-proficient cancer cells in response to PARPi [100,101].

## 5. Reversion Mutations in PARPi Resistance

Researchers investigated the effects of reversion mutations on PARPi resistance in 2008 in a *BRCA2*- deficient cell line and found that the reconstructed *BRCA2*-deficient cells developed PARPi resistance [63,67,91]. Wild-type recessive mutations were restored; the *BRCA2* reading frame is an important intermediary of acquired resistance to platinum and PARPi [91]. Multiple *BRCA* reversion mutations were identified by employing liquid biopsy and cfDNA (circulating cell-free DNA) to re-establish *BRCA1/2* mutations, resulting in PARPi resistance [63,67]. Recently, two reversion mutations were identified—c.4897_6807del and c.4434_5686delinsTT truncated *BRCA2* protein—which were supposed to be capable of causing PARPi resistance [63,67,91,92].

### 5.1. Protection of the DNA Replication Fork in PARPi Resistance

*BRCA1* and *BRCA2* protect newly synthesized DNA at delayed repetition forks from *MRE11*/DNA2-dependent decay [102]. As *BRCA1/2* is damaged, the lack of DNA replication fork protection causes genome instability and cell death—but at the same time, HR is not caused by PARPi resistance in patients with *BRCA1/2* [95,99]. *MRE11*-mediated fork degradation is suppressed by *FANCD2*; FANCD2 is observed in *BRCA1/2*-mutated BC. Overexpression of FANCD2 leads to resistance to PARPi [101,102]. *RADX* deletion reinstates fork protection but not HR, by modifying *RAD51* at replication forks and restoring PARPi sensitivity in *BRCA2*-mutations [96]. Therefore, its strength lies in bringing a novel approach to the future of cancer therapy. Delayed replication divergences are the main foundation of genome variability in multiplying cells, which is essential to maintain cell viability [38,95]. Pathways involved in *ATR/CHK1*-dependent checkpoint activation and RecQ helicases also play essential roles in replication fork protection and genome stability maintenance [48,49]. Therefore, they might function as part of the mechanisms of PARPi resistance. However, there are no relevant preclinical or clinical studies up to now, which are expected to be taken into consideration in the future.

### 5.2. Epigenetic Modification, Restoration of PARylation, and Pharmacological Alteration in PARPi Resistance

PARPi efficacy may be affected by epigenetic modifications; various lines of therapy are received by patients before PARPi treatment, leading to reduced *BRCA* expression and causing PARPi resistance [59]. NHEJ repair is suppressed by MiR-622 and miR-493-5p by altering multiple pathways relevant to genome solidity [31,73,99,100]. Deubiquitination of the *BARD1* BRCT domain by USP15 supports *BRCA1* retention at DSBs and causes PARPi resistance [100,101]. Most of these modifications take place similarly through acetylation of 53 bp1-repressed NHEJ and promotion of HR by undesirable alterations in 53 bp1 recruitment to DSBs, leading to *BRCA1*-deficient cells developing resistance to PARPi. m6A (N6-methyladenosine) plays an important role in PARPi resistance [100,101,102]; increased expression of m6A in PEO1 cells has been established. c-Met mediates the phosphorylation of PARP1 at the position of Tyr907, leading to drug resistance to PARPi [94,103]. *BRCA2*-mutated animal models develop mammary tumors [84,85]. Increased expression levels of P-gp-mediated drug efflux are involved in resistance to PARPi, validated using in vivo and in vitro investigations; this resistance may be reversed by adding regimens of P-gp inhibitors [104]. Long-term overexpression of ABCB1 leads to changes in pharmacokinetic properties and causes PARPi resistance [104].

## 6. Clinical Implications of PARPi Resistance

Effective clinical strategies and increasing PARPi sensitivity may overcome drug resistance [37,49,58,79]. oHSV (PARPioncolytic herpes simplex virus) combination treatments are approved by the FDA for frequent melanoma, and are inherently engineered to selectively kill cancer cells, due to their characteristics of amplifying and spreading within the tumor but not normal tissue [105]. They are actively involved in manipulating DDR [105]. The combination treatment of MG18L with olaparib significantly improved efficacy in both PARPi-sensitive and -resistant GSC-derived tumors [29,56,63,64,65]. MG18L detects proteasomal damage to *RAD51*, explaining glioblastoma stem cells’ (GSCs’) susceptibility to killing by PARPi in a synthetic lethal-like fashion in vivo and in vitro [48,92]. Combination therapy is recommended to enhance the efficacy of PARPi-ionizing radiation (PI) and nuclear localization (NL) [48]. NL is essential for *BRCA1* to contribute to HR-facilitated DNA repair [14]. PARPi encourages radiosensitization in animal models as well as in cell lines; increased radiosensitivity in preclinical model systems was observed due to HR restoration by 53BP1 pathway inactivation [31,37,40,99]. Stimulating HR restoration via 53BP1 pathway inactivation further enhanced radiosensitivity in preclinical model systems [40,99]. It was observed that *BRCA1*-mutated tumors lead to drug resistance due to *BRCA1*-independent HR restoration and are sensitized to radiotherapy [31,37,40]. Clinical investigations (NCT00649207) similarly trying to identify the efficacy of PARPi-IR combination with veliparib in Phase I observed a more clinically effective outcome [40,80,81,82,83]. A randomized, controlled phase IIb study has been carried out. Two other phase I trials (NCT01264432, NCT01589419) indicated that PARPi-IR combination treatment was well-tolerated and showed good responses as well. Patients received veliparib PO BID on days 1–21 (days 5–21 of course 1). Patients underwent LDFWAR in BID on days 1 and 5 of weeks 1–3 [40,80,81,82,83]. Treatment were repeated every 28 days for 3 courses in the absence of disease progression or unacceptable toxicity [40,80,81,82,83]. CDK inhibitor was re-sensitized in TBNC cells with dinaciclib, previously reported as being resistant to niraparib [97,98]. CDK12 is involved in PARPi resistance by inactivating somatic alterations; CDK12 mutations lead to sensitivity to PARPi [97,98]. CDK12 inhibitors are reversed by de novo and attained PARPi resistance in *BRCA1*-mutant BC cells [97,98]. PARPi has also been used in combination with HSP90 inhibitors, *WEE1* inhibitors, and *ATR/CHK1* inhibitors [49]. *HSP90* plays an important role in *BRCA1* function [49]. *HSP90* inhibitor (7-dimethylaminoethylamino-17-demethoxygeldanamycin) reverses the resistance state by decreasing the quantity of *BRCA1* protein. *WEE1* inhibitors and *ATR/CHK1* treatment also play an important role in reversing PARPi resistance [49]. Various clinical trials are going on to evaluate the safety and efficacy of combination therapy for clinical implementation, such as NCT03787680, NCT03330847, NCT03878095, NCT03462342, NCT03428607, NCT03682289, NCT03579316, NCT04197713, NCT02576444, NCT02511795, and NCT04 065269 [3].

## 7. CRISPR/Cas9 in Reverse Mutation

CRISPR/Cas9 technology has facilitated an attractive paradigm shift in gene editing for clinical and therapeutic intervention through the generation of various CRISPR/Cas9-based high-throughput screens [106]. For instance, this technology has been used for the efficient replacement of hotspot mutation regions, which leads to the suppression of tumor progression, reduction of drug resistance, and improvement of drug efficiency [107,108]. CRISPR/Cas9-mediated technology could be used to reverse the resistance factor, by targeting mutations caused by genetic heterogenicity at DNA repair pathways, the BER mechanism, HR, and NEJ deficiency-based repair mechanisms [107]. Experts from the field have enhanced the efficacy of PARPi resistance proteins by using CRISPR/Cas9 [108]. A researcher from the field exhibited that combination of mild hyperthermia, Olaparib, an HSP90 inhibitor, and 17-(dimethylaminoethylamino)-17-demthoxygeldanamycin (17-DMAG) led to a complete loss of tumor growth [3,107,108]. For instance, all of the mice treated with this combination survived during the treatment [107]. On the contrary, the survival of mice who received a treatment of hyperthermia and PARP inhibitors was about 36% [97]. High-throughput analyses of the CRISPR/Cas9-based library are used to analyze mutations for clinical drug resistance with high sensitivity and specificity [108]. The histone acetyltransferase (HAT) enzyme catalyzes the reaction of adding acetyl groups to lysine residues on histone complexes. HDI-treated cells become responsive to PARP inhibition because of the *BRCA*ness effect [47]. First, HDI blocks the deacetylation of the *HSP90* heat shock protein, which leads to hyperacetylation and inhibition of *HSP90* [49,97,98]. As a result, several client proteins of *HSP90* including *BRCA1*, *RAD52*, *ATR*, and *CHK1* cannot interact with it [49,97,98]. In triple-negative breast cancer, treatment of HDI induces *BRCA*ness and inhibits stemness for sensitization to PARP inhibitors [47,49,97,98]. Moreover, recent studies showed that HDI suberoylanilide hydroxamic acid (Vorinostat, SAHA), PARPi, and PJ34, synergistically induce cell death in anaplastic thyroid carcinoma and leukemia cells [54,92]. CRISPR/Cas9-mediated screening has been used to identify candidates involved in paclitaxel-resistant TNBCs [108]. In vitro and in vivo genetic and cellular analyses have elucidated the essential role of the *MITR/MEF2A/IL11* axis in paclitaxel resistance and have provided a novel therapeutic strategy for TNBC patients to overcome poor chemotherapy responses [107,108,109]. Resistance to PARPi greatly hinders therapeutic effectiveness in TNBC; the mechanisms of PARPi resistance, including increased expression of *MDR1*, dissociation of PARP1 and PARG, HR restoration, and restoration of replication fork stalling, all reverse the DNA replication pressure and lower the sensitivity to PARPi treatment [84,94,103,104]. The CRISPR/Cas9-based approach could be useful in generating a knockout or knock-in TNBC genome and reversing drug resistance [107,109].

## 8. Conclusions

TNBC tumors are established by their mutual histopathologic and genetic characteristics. The conventional treatment presently prescribed in hospitals for TNBC depends primarily on the clinical stage of the disease and tolerability to treatment—usually accompanied by corticosteroids (dexamethasone) and drugs to control symptoms (ondansetron, etc.) to reduce adverse effects such as inhibition of DNA and RNA synthesis, inhibition of the topoisomerase II enzyme, generation of free oxygen radicals, induction of histone eviction from chromatin, etc. High-level genomic diversity is shown by *BRCA1*-associated cancers and sporadic triple-negative tumors, which can repair DNA damage. Sporadic TNBC tumors and *BRCA* associated cancers are high grade and have *p53* tumor suppressor gene mutations, overexpression of the epidermal growth factor receptor, and resistance to chemotherapy, ineffective hormonal therapy, and *HER2*-directed therapy, due to the triple-negative nature of the disease. PARP inhibition and platinum compounds are effective treatment options for TNBC with HR deficiency. PARP-based monotherapy is unlikely to encourage cancer cell death in *BRCA*-proficient tumors—the optimal chemotherapeutic option for the future remains the combination of PARP inhibition with either cytotoxic drugs or impairment of mechanisms of DNA repair. In *BRCA*-deficient tumors, monotherapy with a PARP inhibitor is already an effective therapeutic approach that proves the concept of synthetic lethality.

## Figures and Tables

**Table 1:** List of PARP inhibitors.

Compound Name	Compound Structure	Efficacy	IC_50_
Nicotinamide	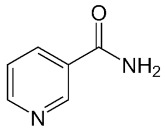	PARP inhibitor and by-product of the PARP reaction; many pharmacological actions other than that of inhibiting PARP	210 μM
3-aminobenzamide	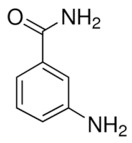	Benzamides are free radical scavengers, among other pharmacological actions	33 μM
PD128763	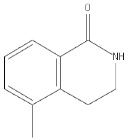	Cytoprotective agent, chemosensitizer, and radiosensitizer; adverse effect of compound causes hypothermia	420 nM
DPQ	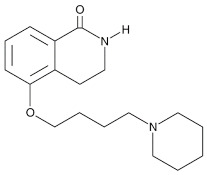	A commonly used Warner–Lambert PARP inhibitor compound based on an isoquinoline core	1 μM
NU1025	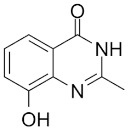	Potentiators of anticancer agent cytotoxicity	400 nM
4-ANI	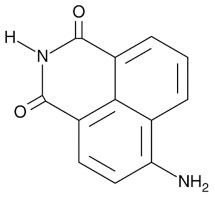	PARP in DNA repair and cell death	180 nM
ISO	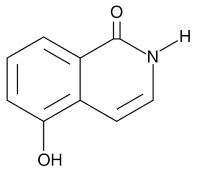	PARP in DNA repair and cell death	390 nM
Olaparib (Lynparza)	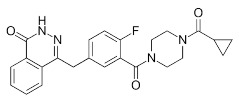	Use in a *BRCA1*-positive patient with metastatic triple-negative breast cancer, without the initial use of platinum-based chemotherapy, showed significant rapid near-resolution of large liver metastasis while patient experienced gout-like symptoms	1 nM
Niraparib (Zejula)	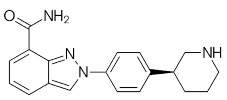	Niraparib in combination with pembrolizumab in patients with triple-negative breast cancer	4 nM
Talazoparib (Talzenna)	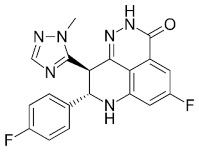	Ferm line *BRCA*-mutant, *HER2*-negative locally advanced or metastatic breast cancer	0.6 nM
Veliparib (ABT-888)	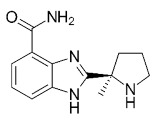	Received orphan drug status for lung cancer	2 nM
INO-1001	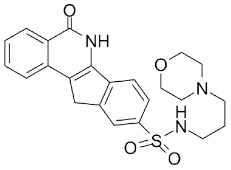	Potent enhancer of radiation sensitivity and enhances radiation-induced cell killing by interfering with DNA repair mechanisms, resulting in necrotic cell death	105 nM
E7449	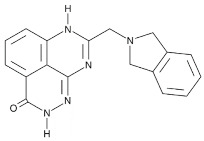	Antitumor activity of E7449; a novel PARP 1/2 and tankyrase 1/2 inhibitor	1 nM
CEP-8983	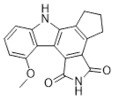	Increases the sensitivity of chemoresistant tumor cells to temozolomide	20 nM
Pamiparib (BGB-290)	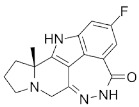	Pamiparib has potent PARP trapping, the capability to penetrate the brain, and can be used for the research of various cancers including solid tumors	0.9 nM
Fluzoparib (SHR-3162)	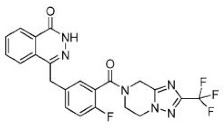	Inhibitor of poly-adenosine diphosphate(ADP)ribose polymerase (PARP) 1/2 being developed for the treatment of *BRCA1/2*-mutant solid tumors.	1.5 nM

**Table 2:** Efficacy of PARP inhibitors.

Name of the Molecules	T_max_ (h)	t (h)	AUC (lgh/ mL)	C_max_ (lg/mL)	CL/F (L/h)	V_z_/F	References
Olaparib capsule formulation 300 mg	1.49 (0.57–3.05)	13.02 (8.23)	55.20 (67.4)	8.05 (24.3)	6.36 (3.47)	112.1 (59.84)	[30]
Olaparib tablet formulation 300 mg single dose (fasted)	1.50 (0.50–5.85)	12.2 (5.31)	43.6 (54.3) [AUC_t_] 43.0 (55.2) [AUC]	7.00 (35.0)	7.95 (4.23)	146 (142)	[32]
Olaparib tablet formulation 300 mg single dose (fed)	4.00 (1.00–12.0)	12.2 (5.31)	46.0 (56.6) [AUC_t_] 45.4 (57.1) [AUC]	5.48 (40.5)	7.55 (3.99)	127 (107)	[32]
Veliparib monotherapy 40 mg (10 mg, fasting)	1.2 ± 0.8	5.9 ± 1.3	2.23 ± 0.82 [AUC_t_] 2.43 ± 1.07 [AUC]	0.36 ± 0.13	19.0 ± 7.36	NA	[34,40]
Veliparib monotherapy 40 mg (10 mg, fed)	1.2 ± 0.7	5.8 ± 1.2	2.45 ± 0.93 [AUC_t_] 2.65 ± 1.17 [AUC_t_]	0.37 ± 0.12	17.3 ± 6.41	NA	
Veliparib monotherapy 40 mg (40 mg, fasting)	1.3 ± 0.9	5.8 ± 1.3	2.24 ± 0.98 [AUC_t_] 2.45 ± 1.24 [AUC_t_]	0.34 ± 0.12	19.5 ± 7.66	NA	
Veliparib monotherapy 40 mg (40 mg, fed)	2.5 ± 1.1	5.8 ± 1.4	2.14 ± 0.80 [AUC_t_] 2.35 ± 1.06 [AUC_t_]	0.28 ± 0.09	19.7 ± 7.51	NA	
Veliparib metabolite M8	2.4 (3.5–9.8)	–	0.3–1.9 [AUC_int_]	0.011 (0.007–0.014)	NA	NA	[34,40]
Niraparib 300 mg/day	3.1 (2.0–6.1)	a	14.117 (AUC24)b	1.921	NA	NA	[12]
Niraparib metabolite: unlabeled M1 plasma	9.02	78.4	41.2 (AUC_t_)	476	NA	NA	[15]

**Table 3:** Clinical Trials of PARP Inhibitors in TNBC.

Name of Drug	Types of Inhibitors	Prior Treatment	Type of Population	Status	ClinicalTrials.gov Identifier
AZD1775 in patent with TNBC LYNPARZATM	PARP Inhibitor, patent with TNBC	Olaparib in combination with AZD6738 mutated (ATM)	Inhibitor of Ataxia-Telangiectasia and *WEE1* inhibitor	Phase II	NCT03330847
AZD1775 in patent with TNBC LYNPARZATM	PARP Inhibitor, patent with TNBC	Olaparib with radiation therapy, after chemotherapy	Inhibitor of ataxia-telangiectasia	Phase I	NCT03109080
AZD1775, LYNPARZATM	Patent with TNBC	Olaparib with atezolizumab	Inhibitor of *PD-L1*	Phase II	NCT02849496
AZD1775, LYNPARZATM	Patent with TNBC	Oolaparib with paclitaxel and carboplatin	Inhibitor of germline *BRCA* mutated	Phase II/III	NCT03150576, NCT02789332
AZD1775, LYNPARZATM	Patent with TNBC	Olaparib with AZD2171 orally	Inhibitor of *VEGFR* tyrosine kinase	Phase I/II	NCT01116648
AZD1775, LYNPARZATM	Patent with TNBC	Olaparib with PI3K inhibitor, BKM120	Inhibitor of BKM120	Phase I	NCT01623349
AZD1775, LYNPARZATM	Patent with TNBC	Olaparib with onalespib	Inhibitor of heat shock protein 90	Phase I	NCT02898207
AZD1775, LYNPARZATM	Patent with TNBC	Olaparib with AZD2014	mTORC1/2 or Oral AKT inhibitor	Phase I/II	NCT02208375
PARP1/2 inhibitor Veliparib	Patent with TNBC	Veliparib in combination with cyclophosphamide	Inhibitor of *EGFR*, *HER2*, *BRCA*, and tyrosine kinase	Phase II and failed in phase III trials	NCT01306032
PARP1/2 inhibitor Veliparib	Inhibitor of tyrosine kinase, *HER2*, and *BRCA*	Veliparib in combination with carboplatin	Patients with TNBC	Completed phase I study	NCT01251874
PARP1/2 inhibitor Veliparib	Inhibitor of *EGFR*, *BRCA*, and tyrosine kinase	Veliparib with vinorelbine	Patients with TNBC	Completed phase I	NCT01281150
PARP1/2 inhibitor Veliparib	Inhibitor of *EGFR*, *HER2*, *BRCA*, and tyrosine kinase	Veliparib with cisplatin	Patients with TNBC	Completed phase I	NCT01104259
PARP1/2 inhibitor Veliparib	Inhibitor of *EGFR*, *HER2*, *BRCA*, and tyrosine kinase	Veliparib with pegylation	Patients with TNBC	Completed phase I	NCT01145430
PARP1/2 inhibitor Veliparib	Inhibitor of *EGFR*, *HER2*, *BRCA*, and tyrosine kinase	Veliparib with pegylation	Patients with TNBC	Completed phase I	NCT01145430
PARP1/2 inhibitor Veliparib	Inhibitor of *EGFR*, *HER2*, *BRCA*, and tyrosine kinase	Veliparib with lapatinib	Patients with TNBC	Phase I	NCT02158507
PARP1/2 inhibitor Veliparib	Inhibitor of *EGFR*, *HER2*, *BRCA*, and tyrosine kinase	Veliparib in combination with irinotecan HCl	Patients with TNBC	Phase I I	NCT00576654
PARP1/2 inhibitor Veliparib	Inhibitor of *EGFR*, *HER2*, *BRCA*, and tyrosine kinase	Veliparib with cisplatin	Patients with TNBC	Phase II	NCT02595905
AZD2281 and Ku-0059436 PARP1/2 inhibitor (Selective)	PARP inhibitor; *BRCA* Mutated	Olaparib alone, or in combination with durvalumab MEDI4736 against PD-L1	*HER2*-negative treated mTNBC	Phase-II	NCT00679783 NCT03544125 NCT02484404 NCT03167619 NCT02681562 NCT02484404
PARP1/2 inhibitor Veliparib	Inhibitor of *EGFR*, *HER2*, *BRCA*, and tyrosine kinase	Veliparib plus carboplatin	Patients with TNBC	Phase III	NCT02032277
Iniparib BSI-201 and SAR240550	Competitive PARP inhibitor; ability to form adducts with many cysteine-containing proteins	Combination with gemcitabine and carboplatin	Patients with TNBC	Phase II	NCT00813956 NCT01045304 NCT01130259
Iniparib BSI-201 and SAR240550	Competitive PARP inhibitor; ability to form adducts with many cysteine-containing proteins	Combination of iniparib with paclitaxel for TNBC compared to paclitaxel alone	Patients with TNBC	Competed phase II	NCT01204125
Iniparib BSI-201 and SAR240550	Competitive PARP inhibitor; ability to form adducts with many cysteine-containing proteins	Iniparib with irinotecan	Patients with TNBC	Phase II trial	NCT01173497
Niraparib	≥1 anti-*HER2* treatment; PARP inhibitor	Niraparib plus trastuzumab IV	Metastatic *HER2*+ breast cancer	Phase Ib/II (recruiting)	NCT03368729
Niraparib	PARP inhibitor	One anthracycline and/or taxane in the (neo-) adjuvant or Niraparib	Advanced/metastatic *BRCA1*- like	Phase-II, Active, not recruiting	NCT02826512
Niraparib	PARP inhibitor	≥1 line of therapy Niraparib plus everolimus	Patients with TNBC	Phase I Recruiting	NCT03154281
Niraparib	Germline *BRCA* mutation-positive (PARP inhibitors)	≤2 prior cytotoxic regimens and Niraparib versus physician‘s choice	Advanced or metastatic breast cancer	Phase III Active, not yet recruiting	NCT01905592 (BRAVO)
Niraparib	Metastatic TNBC inhibitors (PARP inhibitors)	≤2 lines of cytotoxic therapy, Niraparib plus pembrolizumab	Advanced or metastatic TNBC	Phase I/II Active, not yet recruiting	NCT02657889 (KEYNOTE-162)
veliparib	Metastatic TNBC inhibitors (PARP inhibitors)	≤2 lines of cytotoxic Chemotherapy, Carboplatin, and paclitaxel with or without veliparib	Locally advanced unresectable *BRCA* associated	Phase III Recruiting	NCT02163694
veliparib	Metastatic TNBC inhibitors (PARP inhibitors)	Veliparib with temozolomide versus veliparib with carboplatin and paclitaxel versus placebo with carboplatin and paclitaxel ≤2 lines of cytotoxic chemotherapy	Metastatic TNBC	Randomized phase II, Ongoing	NCT01506609
veliparib	Metastatic TNBC inhibitors (PARP inhibitors)	Veliparib versus atezolizumab versus veliparib plus atezolizumab	Stage III–IV TNBC	Randomized phase II Ongoing	NCT02849496
veliparib	Metastatic TNBC inhibitors PARP inhibitors)	Cisplatin and placebo versus cisplatin and veliparib ≤1 line of cytotoxic chemotherapy for metastatic disease	Metastatic TNBC and/or *BRCA* mutation-associated breast cancer	Phase II Active, not recruiting	NCT02595905
veliparib	Metastatic TNBC inhibitors PARP inhibitors)	Temozolomide and veliparib ≥1 chemotherapy regimen	Metastatic TNBC and/or *BRCA* mutation-associated breast cancer	Phase II, Active, not recruiting	NCT01009788
Talazoparib	Neoadjuvant therapy	None	Primary breast cancer ≥1 cm with a deleterious BRCA mutation	Phase II, Active, not recruiting	NCT02282345
Talazoparib	Advanced TNBC and HR deficiency and advanced *HER2*-negative breast cancer or other solid tumors with a mutation in HR pathway genes	≥1 line of therapy	Talazoparib	Phase II, Recruiting	NCT02401347
Talazoparib	Metastatic TNBC inhibitors PARP inhibitors	Platinum-containing regimen with disease progression > 8 weeks	Metastatic breast cancer with *BRCA* mutation	Phase II Terminated (Primary Analysis and study completed Not stopped	NCT02034916 (ABRAZO)
Talazoparib	Metastatic TNBC inhibitors PARP inhibitors	≤3 chemotherapy-inclusive regimens Talazoparib versus physician‘s choice	Locally advanced and/or metastatic breast cancer with germline *BRCA* mutations	Phase III Completed	NCT01945775 (EMBRACA)
Rucaparib	Metastatic TNBC inhibitors PARP inhibitors	≤5 prior chemotherapy Rucaparib regimens in the last 5 years	Patients presenting with metastatic breast cancer (MBC)	Phase II, Completed	NCT00664781
Rucaparib	Metastatic TNBC inhibitors PARP inhibitors	≥1 line of chemotherapy, Rucaparib	Patients with a *BRCA*ness genomic signature	Phase II Completed	NCT02505048 (RUBY)
Rucaparib	Stage I–III patients with TNBC or inhibitors PARP inhibitors	Neoadjuvant chemotherapy Cisplatin with rucaparib	*ER/PR*+, *HER2*- negative breast cancer with known *BRCA1/2* mutations	Phase II Completed	NCT01074970
Rucaparib	TNBC inhibitors	≥3 prior chemotherapy regimens, Rucaparib	Patients with advanced solid tumors with evidence of germline	Phase I/II Active, not recruiting	NCT01482715
Rucaparib	TNBC inhibitors	≤5 prior chemotherapy regimens in the last 5 years, Rucaparib	Patients with MBC carriers of a *BRCA1/2*	Phase II Completed	NCT00664781
Rucaparib	TNBC inhibitors	≥1 line of chemotherapy Rucaparib	Patients with a *BRCA*ness genomic signature	Phase II Completed	NCT02505048 (RUBY)
Rucaparib	TNBC inhibitors	Neoadjuvant chemotherapy Cisplatin with rucaparib	Advanced solid tumors with evidence of germline or somatic *BRCA* mutation	Completed	NCT01074970
Rucaparib	TNBC inhibitors	≥3 prior chemotherapy regimens	Advanced solid tumors with evidence of germline or somatic *BRCA* mutation	Phase I/II Active, not recruiting	NCT01482715
